# Ecosystem Services Approach in Turnicki National Park Planning: Factors Influencing the Inhabitants’ Perspectives on Local Natural Resources and Protected Areas

**DOI:** 10.1007/s00267-024-02016-x

**Published:** 2024-07-18

**Authors:** Mariusz Daniel Boćkowski, Joanna Tusznio, Marcin Rechciński, Małgorzata Blicharska, Arash Akhshik, Małgorzata Grodzińska-Jurczak

**Affiliations:** 1 Institute of Environmental Sciences, Jagiellonian University, Kraków, Poland; 2 Institute of Geography and Spatial Management, Jagiellonian University, Kraków, Poland; 3 Natural Resources and Sustainable Development, Department of Earth Sciences, Uppsala University, Uppsala, Sweden

**Keywords:** Protected area conflict, Participatory planning, Local community, Benefits from nature, Ecosystem management

## Abstract

Despite changing paradigms in nature conservation, protected areas, such as national parks, remain key tools for nature conservation. Today, protected areas are perceived as socio-ecological systems, therefore using an ecosystem services approach may help in their designation. Here, we focus on the planned Turnicki National Park located in the far eastern part of the Polish Carpathian Mountains and conflict between proponents of the park establishment and local stakeholders. We used an ecosystem services-driven questionnaire survey among local communities to analyze interactions between the perception of ecosystem services and opinions about national parks, and the role of social and economic status in shaping these opinions. We found links between opinions towards national parks and other factors: age, life span in a municipality, level of education, and an average net income. Respondents who perceived benefits from nature were more positive towards national parks in general and the Turnicki National Park specifically; however, those who prioritized provisioning services were more skeptical. Also, we distinguished four Fuzzy-Set Qualitative Comparative Analysis models which describe factors shaping opinions on national parks, respectively. The study has shown that the ecosystem services lens perspective can help in exploring the factors crucial while establishing the protected areas in specific social and economic context. The main implication for the study is careful consideration of the role of national park to protect the local environment in harmony with social needs and economic development.

## Introduction

Despite significant efforts undertaken over the last few decades, ecosystem loss, fragmentation and degradation are still ongoing worldwide, including Europe (Steffen et al. 2015, EEA 2021). The pan-European goals of halting biodiversity decline have been realistically not achieved, as planned, either in 2010 or 2020, although the overall ambition of the European Union (EU) biodiversity policy is increasing. Currently, the new EU Biodiversity Strategy (EC 2020) aims to halt the loss and restore biodiversity by 2030. It acknowledges that rich biodiversity and healthy ecosystems are strongly linked to human well-being through the provision of goods and services and contributions to social relations and efficient economies (Harrison et al. 2014, Folke et al. 2021). There is a need to develop new and adapt existing conservation tools for effective protection of such contributions, which also require a better integration of the human dimension perspective into biodiversity conservation (Bennett et al. 2017).

**Fig. 1:**
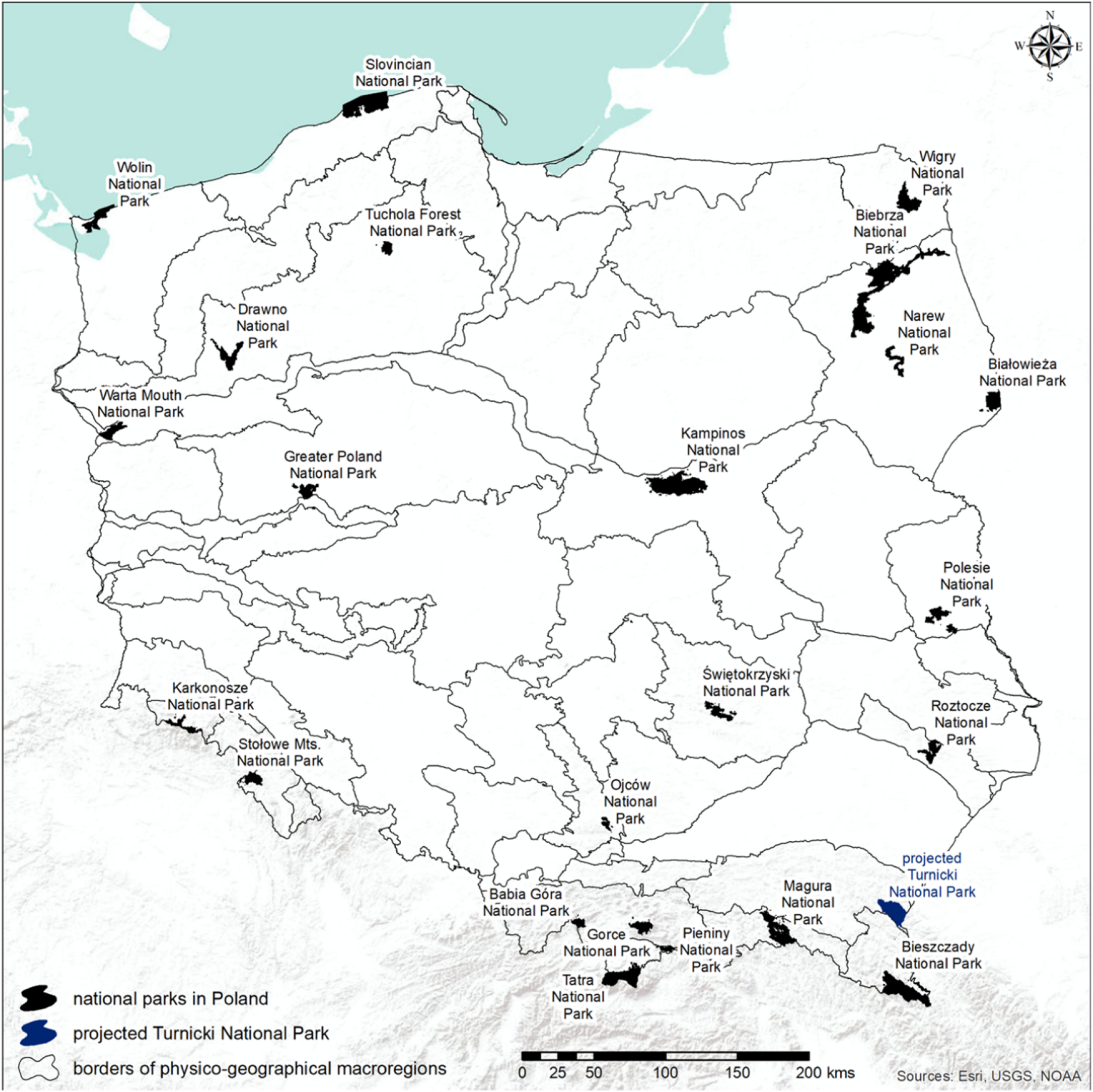
Map of the system of national parks in Poland against the background of physico-geographic regions. Note that the projected Turnicki National Park would be the only national park in Poland in the foothills range

Among various social factors, negative opinions and resulting public resistance towards the establishment and enlargement of protected areas (PAs) are those that hinder conservation efforts (Kati et al. 2014, Mika et al. 2019). Globally, terrestrial PAs are developing very slowly and do not comply with international conservation targets (UN WCMC 2021). On the regional level, it is driven by cross-cutting issues, such as stakeholders’ fears and prejudices regarding the negative impacts of PAs, which resulted, for example, in numerous conflicts over designations of Natura 2000 areas in European Union member countries two decades ago (Paavola 2004; Grodzińska-Jurczak and Cent 2011). National institutional and legal factors add on top of that – for instance, in Poland, not a single NP has been established or significantly enlarged since 2001, due to the national environmental law giving local governments veto decisions on the enlargement or establishment of new NPs in their localities (Niedziałkowski et al. 2014). Since that time, the Polish academia has strongly advocated for at least three new NPs and the enlargement of Białowieża National Park (Szafraniuk et al. 2021, Niedziałkowski et al. 2014). None of these was implemented just due to a lack of consent from local governments. Until today, 23 existing NPs (most of them classified into IUCN category II, 9 of them have status of International Biosphere Reserves) cover only approximately 1% of territory of the country (Fig. 1). NPs hold the highest rank and are the core areas of the Polish system of PAs. According to a provision of the Polish Nature Conservation Act, a NP is defined as “an area distinguished by special natural, scientific, social, cultural and educational values, with an area of not less than 1,000 hectares, where all nature and landscape values are protected”. NPs cover a wide variety of ecosystem types in Poland, ranging from well-preserved diverse forest types through upland and mountain ecosystems to freshwater, marsh and marine ecosystems. However, the NP system lacks a full representativeness in terms of geography and observed natural diversity. Surface coverage alone places Poland below the EU average (UNEP-WCMC and IUCN 2024) and far away from the latest targets of the EU Biodiversity Strategy for 2030, which calls for strict protection of 10% of land, including all old-growth forests (EC 2020). At the same time, the Polish system of PAs has expanded substantially over the last two decades, mostly due to adoption of the Natura 2000 network (which often overlaps with other PAs including all Polish NPs [EC/EEA 2022]). This raises a question of the actual role that NPs may now play in nature conservation among other legal forms of PAs.

Understanding of PAs has evolved over time. PAs have changed from being isolated islands through elements of conservation networks or objects managed alongside areas surrounding them within the so-called “landscape approach” (Palomo et al. 2014). Accordingly, PAs are today perceived rather as socio-ecological systems and framed within the “people and nature” paradigm, which underlines the importance of human culture and institutions co-creating interactions between communities and the environment (Mace 2014). This new framing builds on a previous and more utilitarian way of considering human–nature relationships (“nature for people”) and engages a multidimensional and nonlinear vision of a shared human–nature environment (Sandbrook et al. 2019). While this broadening of the scope of the nature conservation paradigm is intended to ease tensions between conservation objectives and economic activity, practical difficulties in integrating these visions can stimulate even more environmental conflicts. This was already the case for Central and Eastern European countries where post-socialist nature conservation legislation underwent a gradual and conflicting shift towards more participatory PA management along with the Europeanisation process (Niedziałkowski et al. 2012, Yakusheva 2019).

Here, we claim that exploring such conservation issues through the lens of a socio-ecological system approach may clarify the diverse background of emerging opposition towards the creation or enlargement of PAs. We also believe that the approach can turn respondents’ attention from perceived hindrances associated with PAs towards the benefits of protected nature. Thus, it helps distinguish whether there is a real competition for resources in a given area or there are rather more indirect factors influencing PA perception, such as employment or management (see Allendorf 2022). In light of increasing demands on PAs and their ability to deliver conservation and development benefits simultaneously, global initiatives aim to support socio-ecological system thinking in PAs and mitigation of competition for resources. UNESCO’s World Heritage Sites and Biosphere Reserves have the potential to connect social and ecological parts of the PA system via a multi-scale approach to environmental governance, enhanced local participation, prospective planning and stringent PA zoning (Job et al. 2017). A variety of alternative conservation measures designated as “Other Effective Area Conservation Measures” (OECMs) seek solution to concurrent achieving conservation, socioeconomic and other relevant goals from conventional protected areas (Botha et al. 2021). Importantly for the Central and Eastern Europe, OECMs could also facilitate the achievement of the global 30 × 30 target for ecosystem restoration and conservation under the Carpathian Biodiversity Framework (CBD 2023).

The socioecological systems perspective resonates also within the well-established debate of the park–people relationships (PPR) (e.g., Zube and Busch 1990; McCleave et al. 2006, Allendorf 2022). PPR research has explored multiple aspects of PAs’ human dimensions, such as local history, social and economic context, residents’ relationships with the park staff, people’s physical interaction with nature (e.g., extraction, recreation), their knowledge, perception, behavior, and attitude towards the park (Allendorf 2010, Bragagnolo et al. 2016). The PPR debate may also draw from psychological theories such as the theory of planned behavior (Ajzen 1985) or theory of psychological reactance (Brehm 1966); also, when explaining opposition towards NPs (Schenk et al. 2007). While this variety of explanatory variables is used in different research on PPR in a particular national context, the role of opinions and perceptions towards nature protection is crucial in explaining support or opposition towards establishment or management of NPs (Schenk et al. 2007, Stern 2008, Dimitrakopoulos et al. 2010, Lehnen et al. 2022).

We propose a further extension of the PPR research study to include stakeholders’ perceptions of how nature and NPs contribute to their well-being through the lens of the socio-ecological system approach. It complements the studies on the influence of general environmental values and the perceptions of PAs (e.g., Pietrzyk-Kaszyńska et al. 2012, Dická et al. 2020, Aastrup et al. 2021, Bishop et al. 2022) by providing insight into perceptions of nature that are specific to local environmental and social conditions. At the same time, we acknowledge that any approach applied to local level could not function without broader global change framing. Cross-scale analysis of issues impacting PAs, along with guidance on the interaction between global and local factors, is necessary to make sustainable decisions. For example, climate change impacts on local biodiversity need to be integrated into NP planning, or population growth and demographic change should be considered in the planning of park visitation (Botha et al. 2021; Becken and Job 2014).

Perceived benefits from protected nature are parts of socio-ecological systems that can be conceptualized using the framework of nature’s contributions to people, or more commonly, the ecosystem services (ES) concept (MEA 2005). The concept classifies benefits from nature essential to humans into main categories, such as provisioning, cultural and regulatory/supporting. It also establishes the relationship between biodiversity, ecosystem functions and human well-being (Haines-Young and Potschin 2018). Based on that, the ES lens can be applied to a variety of quantitative and qualitative studies of social and ecological character as well as to nature management practice (Haines-Young and Potschin 2018, see Appendix 3).

Based on previous studies one can hypothesize the linkage between perceptions of ES, opinions and intention to engage in pro-environmental behavior (Lima and Bastos 2020, Lehnen et al. 2022, Tiebel et al. 2021). In our study, we selected explanatory variables based on the contemporary understanding of PPR (Bragagnolo et al. 2016). Accordingly, studying PPR can be successfully performed within the rather resource-oriented framework of ecosystems services by, for example, referring to general opinions towards NPs (Arnberger and Schoissengeier 2012), looking for win-win scenarios (Allendorf and Yang 2013) or planning new NPs (Nastran 2015). Moreover, we observe that the number of studies on perceptions of benefits and losses related to designation of new NPs is still limited.

In our study, we focused on a planned Turnicki National Park (TuNP) in the far eastern part of the Polish Carpathian Mountains. This territory has been, from the 1980s until now, an arena of conflict between proponents of park establishment and local stakeholders opposing that policy. Moreover, in contrast to the world-known Polish case of the enlargement of the Białowieża National Park (Blicharska and Van Herzele 2015; Niedziałkowski 2016), it has never been a subject of investigation from the socio-ecological system perspective, albeit the highest biodiversity values of its old growth forest. The area of planned TuNP serves as an excellent testing ground for exploring whether the ES concept may contribute to planning NP or reforming the concept of NP by encouraging new perspective to existing debates among stakeholders. Specifically, there is a gap in knowledge on the perceived priority of ES delivered from the area of the planned TuNP, which is especially important in cases when the establishment of an NP would change the rules of natural resource use, such as timber production, hunting or tourism. Consequently, we propose to study stakeholders’ perception of limitations and threats to these priority ES, including the role of the proposed NP. Perception of benefits and losses are potentially important explanatory variables for opinions about NPs (Allendorf et al. 2012, Sirivongs and Tsuchiya 2012, Aastrup et al. 2021, He and Su 2022), however have not been linked so far with prioritizing ES for local wellbeing. To address more than stakeholders’ perception, those factors need to be analyzed against other social determinants relevant to the local context, the role of natural resources for local livelihoods and linkages between the local economy and forestry (Boćkowski et al. 2020).

The main goal of our study is to examine the use of the ES lens in explaining opinions towards the planned NP among local inhabitants. Therefore, our analysis is focused on three detailed goals: (1) to analyze the interactions between perception of benefits from nature and opinions towards NPs, (2) to explore how social factors shape opinions about NPs and (3) to discuss potential roles and ideas for an NP for better coexistence with local societies.

## Methodology

### Study Area

**Fig. 2:**
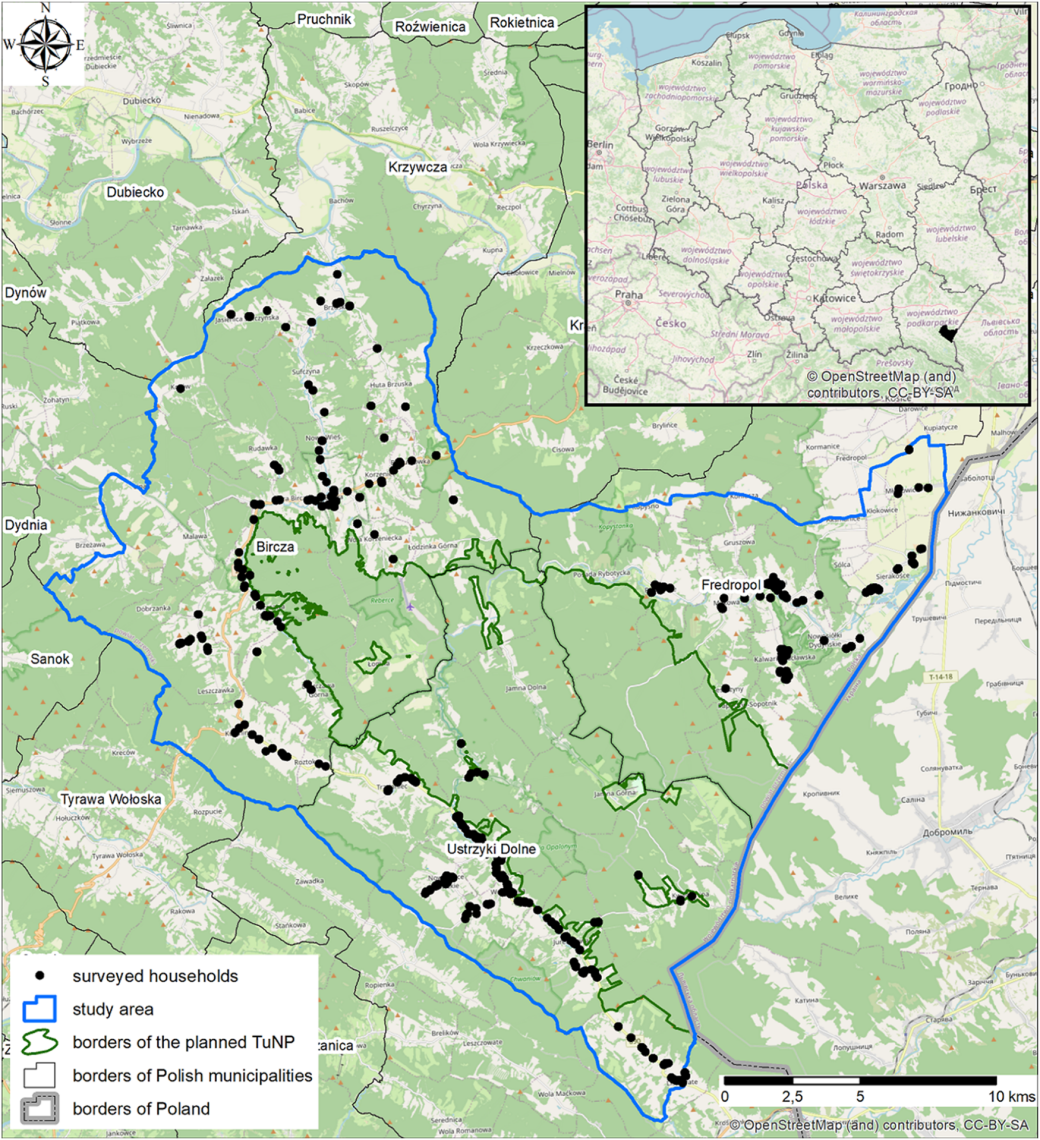
Map of the study area

We collected data on the perception of benefits from nature and opinions towards NPs among respondents residing three municipalities: Bircza, Fredropol and Ustrzyki Dolne, overlapping with the area of the planned TuNP (Fig. 2). Investigated municipalities have the legal right to veto the establishment of the park and they currently use it. Their land includes vast forests located in Subcarpathian Province to the south of Przemyśl at the present border with Ukraine, which has been attracting the attention of naturalists since the 1930s. The area is the last large and relatively untouched forest complex in the Polish foothills including the old-growth trees, rich and unique flora, and fauna (Boćkowski et al. 2020).

The studied area remained largely uninhabited and poorly developed because of World War II hostilities and evictions of the local population that happened shortly after. Then, from the 1960s to the 1980s, it was incorporated into a secret resort for hunting dignitaries, and thus has retained its isolation and remoteness until today (Affek 2015). Currently, the south-eastern part of the province where the municipalities are located represents the most peripheral and economically the least developed part of the region with relatively high registered unemployment ranging from 12.5% (Przemyśl district) to 13.5% (Bieszczady district) compared to an average of 5.2% in Poland (GUS 2019). The Przemyśl district, which includes the municipalities of Bircza and Fredropol, was ranked last (25th) in the provincial entrepreneurship ranking in 2019 in terms of the number of national economy entities per 10,000 population. The local communities are struggling with problems typical for intense transformation and modernization processes, such as: lack of jobs related to the abandonment of agriculture, dependence on social welfare, risk of poverty and exclusion, shortages in infrastructure, low availability of public services and limited resources in terms of human and cultural capital that hinder adaptation to changing labor markets low levels of entrepreneurship and relatively low institutional organization.

The prevailing land use is agriculture and forestry, with a dominant share of forest areas being managed by the Polish State Forests (Annex No. 1). Agricultural land is very fragmented in private holdings, where plots of several hectares dominate. The best conditions for agricultural activity based on microclimate, soil valuation class and terrain prevail in the Fredropol municipality. By contrast, the municipalities of Bircza and Ustrzyki Dolne are much more mountainous and have a nearly 60% forest cover. These all make them ideal for limited husbandry and breeding of animals and other extensive forms of use, such as permanent meadows and pastures.

The municipalities in question are covered by various, less rigid forms of PAs, which, combined with a unique cultural landscape and low population density, makes them attractive for ecotourism and agritourism. Only one of the communes, Ustrzyki Dolne, has a considerably developed tourist infrastructure in the form of hotels, agritourism farms, sports and recreational facilities. While employment related to forestry remains an important part of the local labor market, the structure of entrepreneurship has changed over the last two decades, with a growing share of entities related to trade, construction, and various services.

### Methods

The in-person questionnaire survey (PAPI) was conducted between July and October 2019, after a pilot study in a neighboring area. Address points marking residential buildings for the survey were drawn using a publicly available GIS spatial database. A proportional lottery was used for the five largest settlements, which included at minimum 50% of all address points in each municipality. The remaining points were drawn from over the rest of the study area.

The survey was conducted with one adult in each household and in multi-family buildings with up to 10 apartments. During data collection, we controlled for the age and sex of the respondents to comply with the actual demographical structure of the population. To avoid overrepresentation of any demographic and social group, we rotated the time of day when conducting interviews.

A total of 527 survey responses were collected (response rate of 39.4%) in three municipalities (Table 1). The majority of respondents had moderate levels of education and were either retired or contract workers. While there was a significant group of unemployed or economically inactive respondents, many of them declared during the survey interview that they worked on their own farms.

**Table 1:** Basic sample characteristics

Number of respondents (Bircza/Fredropol/Ustrzyki Dolne)	527 (184/176/167)
Gender in % (men/women)	40.9/59.1
The average age /median	51/54
Minimum/maximum age	18/86
Dominant age (range)	60–64
Years of residence in a municipality (average/minimum/maximum)	41.5/1/86
Average monthly net income per person in a household in US $/national avg.	375/450*
Education in % (lack/vocational/secondary/higher)	(17/27/35/19)
Professional status in % (unemployed/contract/own enterprise/retired)	(14/34/14/35)

* - in 2019

The questionnaire consisted of 34 questions, starting with selection of five key benefits from nature for the local community, based on a modified ES CICES classification (Haines Young and Potschin 2018) and notions adapted to a Polish context and verified in previously conducted research (Pietrzyk-Kaszyńska et al. 2022, Tusznio et al. 2020). The next questions concerned the perceived current limitations and potential threats to delivery of prioritized benefits, respondents’ general opinions towards NPs – first, in general and second, particularly about the planned TuNP. The latter included opinions on potential benefits and losses from its’ creation, and finally level of approval for TuNP. The last sections focused on respondents’ occupational ties to agriculture, tourism and the logging industries, and sociodemographic information.

### Statistical Analysis

The questions about the benefits of nature were analyzed using descriptive statistics and the chi-square test to assess the differences in the prioritization of benefits from nature. The degree of approval for NPs was analyzed using eight statements measured on a Likert scale (Cronbach’s α = 0.844, 48.2% of the total variance explained), reflecting declared positive and negative opinions about the various roles of NPs in the current system of nature conservation. Opinions were derived from the current discourse on NPs in Poland, based on qualitative inquiry in Polish grey literature, media, scientific articles. Due to the lack of normality in the distributions of variables, individual responses against selected independent variables (questions on the scale) were analyzed using the non-parametric test (Kruskal–Wallis) and post hoc pairwise analysis. Then, after reversing the coding of items (2, 4, 6, 8) that negatively loaded the assumed cognitive construct (declared approval for national parks), a synthetic indicator was created (variable “Role of parks”) that represented the overall approval of the current role of NPs. Its’ distribution was non-normal; however, due to the small skewness of the distribution for the created variable (SKE = −0.121, SE_SKE_ = 0.106), one-way analysis of variance (ANOVA) and post hoc analysis (Tukey’s test) were used to examine the influence of the municipality (Annex No. 1) as a differentiating factor. Subsequently, the correlations (Spearman’s ρ) between the synthetic indicator of approval and selected quantitative variables were examined. Specific opinions (approval) towards establishing the TuNP among the municipalities were analyzed using one-way ANOVA. We also examined correlations (Pearson’s ρ) between the opinion on planned TuNP with quantitative variables and the synthetic indicator of approval for NPs, as well as gender-based differences (Student’s t-test) and on the declared professional situation (Kruskal–Wallis test).

**Fig. 3:**
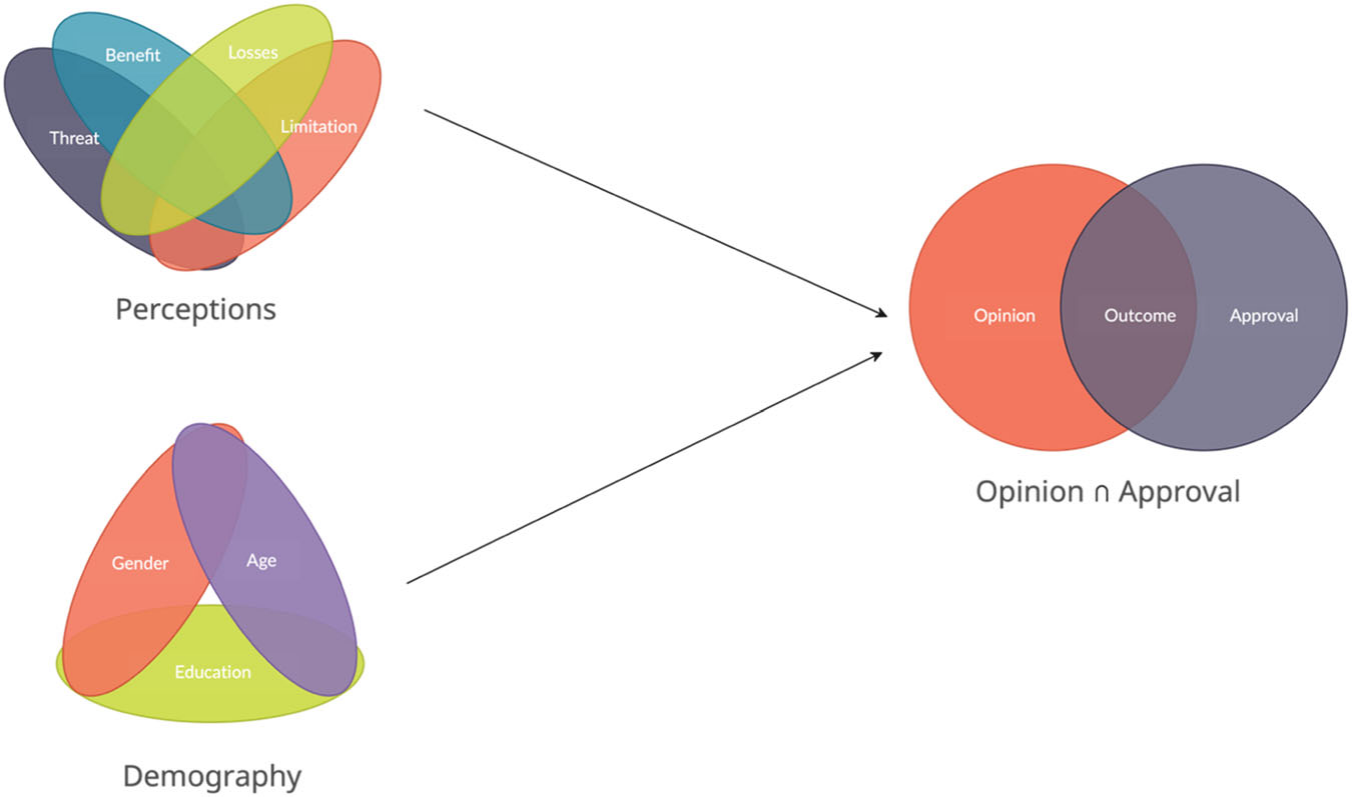
Presenting the intersection of the outcome variable and the recipes. $f$*(opinion* ⋂ *approval)* = *[threat, benefit, losses, limitation, gender, age, education]*

Further, we employed Fuzzy-Set Qualitative Comparative Analysis (fsQCA) to investigate the intricate relationships between various factors with the local inhabitants’ approval of TuNP and their general opinion (synthetic indicator of approval) toward NPs. The Quine-McCluskey algorithm was utilized to perform the fsQCA (See Annex. No. 2 for the details). The factors incorporated into our model were age, education, perceived limitations, perceived threats, perceived benefits, perceived losses, and gender (see Fig. 3).

## Results

### Priority Services From Nature and Perceptions of NPs and TuNP

Benefits from nature of a material character were most frequently selected by respondents as priority services for local wellbeing (52.8% of all choices), (Table 2). Drinking water, wood for heating purposes, harvesting plants and animals constituted up to nearly 40% of all choices. The rest was divided fairly equally between cultural (24.7%) and regulatory services (22.5%).

**Table 2:** The summary of the selected priority services from nature (all 5 choices summed up)

	Count	% of total	% cumulated
Sourcing drinking water (P)	345	13.6	13.6
Sourcing wood for heating purposes (P)	252	9.9	23.5
Harvesting plants (P)	213	8.4	31.9
Animal farming (P)	195	7.7	39.5
Landscape values (C)	189	7.4	47.0
Picking up wild mushroom/sherbs/berries (P)	182	7.2	54.1
Purifying water/air (R)	159	6.3	60.4
Flood and drought protection (R)	144	5.7	66.0
Practising sport/tourism/recreation (C)	124	4.9	70.9
Habitat of wild pollinators (R)	123	4.8	75.7
Spiritual or religous values (C)	91	3.6	79.3
Ecological education (C)	76	3.0	82.3
Mitigating climate extremes (R)	67	2.6	84.9
Noise protection (R)	64	2.5	87.5
Sourcing water for farming/industrial purposes (P)	61	2.4	89.9
Cultural heritage (C)	56	2.2	92.1
Sourcing wood for non-heating purposes (P)	53	2.1	94.1
Inherent value/value of being (C)	51	2.0	96.1
Scientific research (C)	40	1.6	97.7
Sourcing wild animals and derivatives (P)	27	1.1	98.8
Erosion control (R)	16	0.6	99.4
Extracting clay/gravel/minerals (P)	12	0.5	99.9
Other	3	0.1	100.0
Total	2,543	100.0	

*P* provisioning, *C* cultural, *R* regulatory ecosystem services

**Fig. 4:**
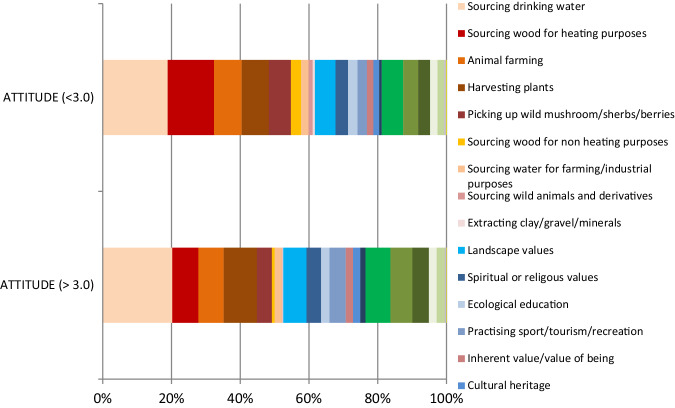
Weighted choice of benefits for respondents who scored >3.0 (“positive”) and ≤3.0 (“negative”) on a 1.0—5.0 scale on “approval of the idea of NPs.” Notes: The weights were assigned as follows: 1st choice = 5 pts., 2nd choice = 4 pts., 3rd choice = 3 pts., 4th choice – 2 pts., 5th choice – 1 pts. Shades of red/orange indicate benefits classified as provisional ES, shades of blue = cultural services, and shades of green = regulatory services; number of “positive” respondents: 1st choice *N* = 277, 2nd choice *N* = 277, 3rd choice *N* = 275, 4th choice *N* = 269, 5th choice *N* = 251; number of “negative” respondents: 1st choice *N* = 245, 2nd choice *N* = 246, 3rd choice *N* = 245, 4th choice *N* = 236, 5th choice *N* = 222

The priority benefits from nature significantly differed among respondents with different levels of “approval for the idea of NPs” (Chi^2^ = 22.2879, *p* < 0.001). The respondents who were negative towards NPs valued provisional ES (57% of all choices) more frequently than respondents positive towards NPs (48%; Fig. 4). Cultural benefits were more important for respondents positive towards the NPs (28%), than those with negative stances (21%).

The share of prioritized provisional, cultural, and regulatory services significantly varied (Chi^2^ = 24.9864, *p* < 0.001) between the three groups of respondents with different opinions (i.e., negative, neutral, or positive) towards TuNP (Annex No. 3). Nevertheless, the respondents appeared to value provisional ES the most regardless of their opinions towards the creation of the park (Annexes No. 4–6).

Significant differences were also found in approval of TuNP between respondents choosing different key benefits (Kruskal–Wallis test H = 43, 373; *p* = 0.004). The respondents who chose sourcing wood for non-heating purposes as a key benefit scored significantly lower in terms of approval of TuNP than respondents who chose habitat for wild pollinators and practicing sports, tourism, or recreation.

### Opinions on National Parks and TuNP Across Studies Municipalities

**Fig. 5:**
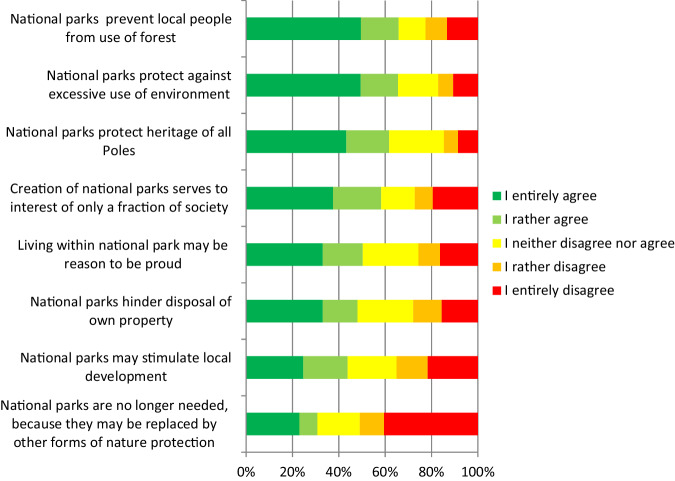
Opinions on statements concerning the relationship between humans and national parks in the entire area of the study

The respondents presented balanced perspectives over various roles of NPs, with no extreme opinions over any statements (Fig. 5). Most of the respondents (65.6%) agreed that NPs prevented excessive exploitation of the environment. Moreover, 65.8% indicated difficulties in using the forest caused by the presence of NPs. More than half of them (61.2%) agreed that NPs protected the heritage of citizens, while 58.3% believed that they served the interests of only a fraction of society. By contrast, 51% did not agree that the existence of NPs was no longer needed.

For nearly all the items in Fig. 5 (except the statement that NPs protect against excessive use of the environment), we found statistically significant differences in responses between respondents from individual municipalities. In multiple pairwise comparisons, respondents from the Fredropol municipality showed greater support than respondents from the Bircza and Ustrzyki Dolne municipalities for the following statements: NPs may stimulate local development (*p* = 0.003; 0.028), protect the heritage of citizens (*p* = 0.007; 0.024) and that living within an NP may be a reason to be proud (*p* < 0.001). On the other hand, respondents from the Fredropol municipality showed significantly lower support than respondents from other municipalities for statements that NPs serve the interests of only a fraction of society (*p* = 0.002; *p* < 0.001) and that NPs may be replaced by other forms of nature protection (*p* = 0.003; *p* < 0.001). Respondents from Bircza municipality showed greater support for the statement that NPs prevented local people from using the forest (*p* < 0.001) than the respondents from the Fredropol municipality, while the latter showed significantly greater support than respondents from both other municipalities for the statement that NPs hinder disposal of own property (*p* < 0.001; (p = 0.023).

**Fig. 6:**
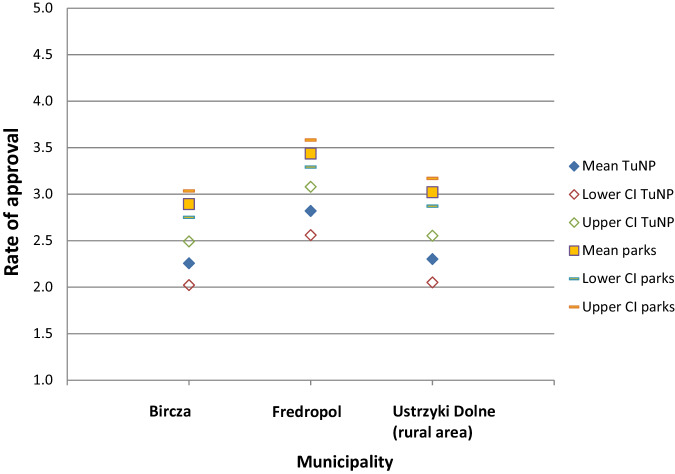
The degree of approval for national parks in general and planned TuNP in the three surveyed municipalities. Note: Measured on a scale of 1–5 where 1 is strongly negative, 3 is neutral and 5 is strongly positive. The overall mean for all municipalities: national parks = 3.12, Turnicki NP = 2.44

There were significant differences among the three municipalities regarding the synthetic indicator of approval towards NPs (*F* = 14.912, *p* < 0.001). It should be noted, though, that the effect size of the examined factor was small (partial ŋ2 = 0.054). Post hoc analysis (Tukey’s test with correction for multiple comparisons) showed significant differences for the pairing of Fredropol–Bircza (*p* < 0.001) and Fredropol–Ustrzyki Dolne (*p* < 0.001), which suggested that the inhabitants of the Fredropol municipality had a more positive opinion towards NPs (Fig. 6).

Of the 527 respondents, 69.4% had heard of the planned TuNP, and they were asked to express their opinion towards its’ creation. In general, respondents from all municipalities were rather sceptical towards the plans to establish the park (Fig. 6). There were significant differences between municipalities (*F* = 5.881, *p* < 0.003), while the effect size for the municipality variable turned was small (partial ŋ2 = 0.031). Respondents of Fredropol demonstrated a more positive opinion towards TuNP than the other two municipalities (*p* < 0.005 for Fredropol–Bircza and *p* < 0.014 for Fredropol–Ustrzyki Dolne).

**Fig. 7:**
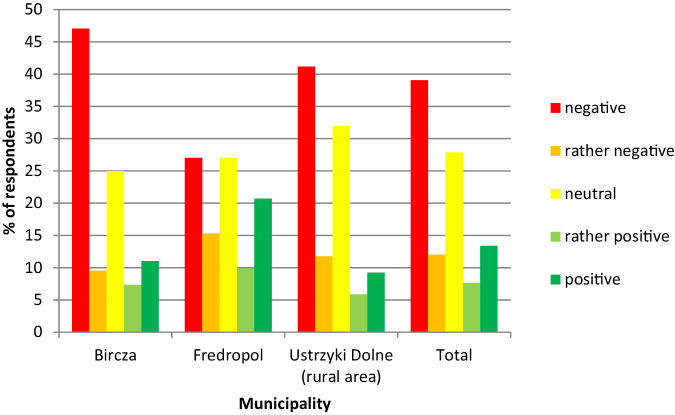
Share of responses to particular categories of approval for Turnicki National Park in the three surveyed municipalities

On average, 28% of respondents had “neutral” opinions regarding the approval of the TuNP, with differences between municipalities, ranging from 25% (Bircza) to 32% (Ustrzyki Dolne) (Fig. 7).

### Opinions on national parks in general and Turnicki National Park against sociodemographic and economic variables

The approval of NPs was weakly negatively correlated with the age of the respondents (Spearman’s ρ = –0.155, *p* < 0.001) and number of years of living in the municipality (*ρ* = –0.299, *p* < 0.001). Contrary, a higher level of education and an average monthly net income per person in a household had a slightly positive effect on support for NPs (*ρ* = 0.214 *p* < 0.001, and *p* = 0.157 *p* = 0.002, respectively). No significant correlation was found between the perceived role of parks and the number of people in a household, or gender (*t* = 1.166; *p* = 0.244.). In case of the occupational situation, the results were inconclusive (Kruskall—Wallis test H = 9.506; *p* = 0.05; results of post hoc analysis in pairs – *p* > 0.05; Annex No. 7).

Similarly to approvals of NPs in general, the positive opinion towards the establishment of the TuNP was weakly negatively correlated with the age of the respondents (*ρ* = –0.104, *p* = 0.046) and the number of years of living in the municipality (*ρ* = –0, 244, *p* < 0.001). The level of education was moderately positively correlated with the opinion towards TuNP (*ρ* = 0.296, *p* < 0.001), as well as with the average monthly net income per person in a household (*ρ* = 0.176, *p* = 0.004). There was no correlation between approval for the establishment of TuNP and the number of people in the household, gender, and the current professional situation. Additionally, there was a strong positive correlation between the opinion towards the creation of the TuNP and opinion on the current role of NPs (*ρ* = 0.705; *p* < 0.001) (Annex No. 7).

### Type of activity versus general opinions on national parks and planned Turnicki NP

Ownership of a farm had a significant negative relationship with positive opinions towards NPs, i.e., farm owners tended to be more skeptical about NPs in general. At the same time, there was no relationship between farm ownership and opinion towards planned TuNP (Annex No. 7). By contrast, respondents related professionally to the tourist sector had more positive opinions both on TuNP and parks in general. Respondents belonging to the broadly perceived logging industry had negative opinions towards parks in general, however, belonging to logging industry was not significantly related to particular opinions towards TuNP (See Annex. No. 8 for the details).

### Perception of the losses, benefits, limitations, and threats from the planned Turnicki NP

Perceived current limitations to the benefits from nature showed no effect on opinions towards NPs and planned TuNP (Annex No. 9). Respondents who saw future threats to benefits from nature were more likely to approve the role of NPs (mean score of approval: 3.19 to 2.97). However, no such relation was found regarding the opinions towards planned TuNP (Annex No. 9).

The majority of respondents perceived losses rather than benefits from the potential establishment of the TuNP (Table 3). Those who saw benefits perceived NPs, including TuNP, positively. Similarly, perception of losses was correlated with more negative opinions on NPs and the planned TuNP.

**Table 3:** Effect of perceived benefits and losses from the creation of Turnicki National Park on opinions of national parks

	Can you see potential benefits from the creation of TuNP?	N	Mean	SD	*t*	Sig. (two-tailed)
Approval for NPs	No	264	2.65	0.89827	−11.230	0.000*
	Yes	102	3.78	0.77474		
Approval for TuNP	No	264	1.94	1.129	−13.399	0.000*
	Yes	102	3.75	1.224		

	Can you see potential losses from creation of TuNP?	N	Mean	SD	*t*	Sig. (two-tailed)
Approval for NPs	No	70	3.79	0.90778	8.324	0.000*
	Yes	296	2.77	0.92382		
Approval for TuNP	No	70	3.79	1.215	9.984	0.000*
	Yes	296	2.13	1.260		

### Pathways towards positive perception of NPs and approval for TuNP – the result of fsQCA

The parsimonious solution derived from the fsQCA revealed four configurations that were significantly associated with the combination of the outcome for opinions towards national parks (NPs) in general and approval of the proposed TuNP (Opinion $\cap$ Approval). The solution coverage was 0.80, indicating that these configurations accounted for approximately 80% of the instances where approval was observed. The solution consistency of 0.55 suggest that these configurations were associated with approval in about 56% of the cases (Table 4).

**Table 4:** Results of fsQCA – configurations of variables leading to the highly positive opinion towards NPs and approval of TuNP

Configuration	Raw Coverage	Consistency
~Losses	0.486516	0.71618
Benefit	0.621779	0.682374
Limitation*~Threat	0.213328	0.601452
Age*Education*~Limitation*~Gender	0.238663	0.747647

Each row in the table represents a configuration that is associated with the intersection of approval of the proposed Turnicki National Park (TuNP) and opinion towards national parks (NPs) in general; ~: indicates the absence or negation of that variable, e.g. ~Gender indicates “not being female”;

*indicates an interaction between those variables; “Raw Coverage” refers to the proportion of all cases with the outcome (approval of the proposed TuNP ⋂ opinion towards national park) that are covered by a given configuration; “Consistency” measures the degree to which a configuration leads to the outcome

The four configurations derived from the fsQCA (Table 4) can be interpreted as follows:

#### Model 1. Absence of perceived losses (~Losses)

This configuration suggests that when local inhabitants do not perceive any losses associated with the establishment of the TuNP, they are more likely to approve the proposed park and have better opinion towards NPs in general (approval of the proposed TuNP ⋂ opinion towards national parks). This could be interpreted as the absence of perceived negative impacts, such as restrictions on land use or loss of access to the natural resources, leading to more positive opinions towards the parks.

#### Model 2. Presence of perceived benefits (Benefit)

This configuration indicates that when local inhabitants perceive benefits from the establishment of the TuNP, they are more likely to approve the proposed park and have a better opinion towards NPs in general. These benefits could include ecosystem services provided by the park, such as recreational opportunities or preservation of biodiversity.

#### Model 3. Presence of perceived limitations and absence of perceived threats (Limitation*~Threat)

This configuration suggests that when local inhabitants perceive limitations associated with sourcing benefits from nature but do not perceive any threats from the establishment of the TuNP, they are more likely to approve the proposed park parks and have a better opinion towards NPs in general. This could be interpreted as local inhabitants recognizing the need for conservation measures (due to perceived limitations) and not seeing the park as a threat to their interests.

#### Model 4. Interaction of age, education, absence of perceived limitations, and absence of gender (Age*Education*~Limitation*~Gender)

This configuration indicates a more complex relationship. It suggests that older and better educated individuals who do not perceive any limitations associated with sourcing benefits from nature and who are not female are more likely to approve the proposed park and have better opinion toward NPs in general. This could be interpreted as older, more educated individuals possibly having a greater appreciation for conservation efforts, and the absence of perceived limitations and gender factor (here not being female) possibly reflecting different cultural or social factors shaping opinions towards the park.

## Discussion

This study contributes to the ongoing discussions in conservation social science on 1) factors influencing people’s perception towards NPs (Schenk et al. 2007, Rossi et al. 2015, Job et al. 2021) and 2) potential contributions of the ES concept to studying and managing perceptions of local communities towards NPs and PAs in general (Martín-López et al. 2012, Brown and Fagerholm 2015, Mączka et al. 2019, Tusznio et al 2020). We found important associations between general opinions towards national parks and various factors. The main variables associated with differences here are related to age, the length of residence in a certain municipality, level of education and net income, which is in line with other studies (Mensah et al. 2017, Ward et al. 2018). Similar to the world-known case of enlargement of the Białowieża Forest (Niedziałkowski et al. 2014), inhabitants of studied municipalities, which are also highly forested areas, seemed to value provisional ES most, although regulatory and cultural services were not by any means neglected. Such a prevalence of appreciation for provisional services was also observed in other studies of ES in planned PAs (He et al. 2018a). This may be due to the underdevelopment of nature-based tourism in an area of not-yet-designated as an NP. Deriving economic benefits from protected areas in a socially balanced way is challenging, and ecotourism could provide a relatively broad distribution of benefits to the local community (Brenner and Job 2022), potentially leading to a higher valuation of cultural and regulatory ES in the future (Zorondo-Rodríguez et al. 2024). However, the contribution of tourism to the local economy is not directly visible and requires detailed studies and tourism monitoring (Job et al. 2021; Spenceley et al. 2021), which should be established within NP structures.

Most notably, respondents who saw benefits from nature were more positive towards NPs (including TuNP). Moreover, respondents who gave priority to provisioning services were more skeptical towards NPs (including TuNP), while approval for the PAs was associated with appreciation of cultural and regulatory services. This corresponds with the interpretation of Blicharska and Herzele (2015), who distinguish three narratives when speaking of the people–forest relationship: “managerial”, “livelihood” and “primeval”. The second one, presented by local inhabitants, underlines the provisional role of the forest in fulfilling people’s needs, whereas “primeval”, presented by NPs supporters, focuses on intrinsic values and natural ecological processes. Perception of general threats to the benefits from nature was associated with a more positive opinion towards NPs and TuNP, predicting losses from creation of TuNP resulted in skepticism towards it. This suggests that to promote positive views on PAs, one needs to address the underlying fears and prejudices, stereotype thinking and build a positive image of PAs as providers of benefits or protectors against threats (Martín-Lopez et al. 2012).

The results suggest that both individual characteristics (age, education, gender) and perceptions of the proposed TuNP (benefits, limitations, threats, losses) play a significant role in shaping opinions (He et al. 2018a). Notably, the absence of perceived losses and the presence of perceived benefits were both associated with an intersection of opinion on NPs in general and approval for the planned TuNP, suggesting that perceptions of the potential positive and negative impacts of the park are crucial factors (Bennett 2016; Job et al. 2021). The interaction of age, education, absence of perceived limitations, and being a male also suggests that these factors may work together in complex ways to influence general and specific opinions on NPs (Pietrzyk-Kaszyńska et al. 2012). The fsQCA allowed us to uncover these complex, non-linear relationships, highlighting the value of this method for understanding opinions towards conservation efforts. The asymmetric nature of the relationships identified in this study underscores the importance of considering multiple non-linear, interacting factors when studying various perceptions towards NPs, contributing to uncovering the complexity of issues in designating new NPs.

General opinions on NPs in our study were mixed, including both advantages and disadvantages. However, respondents doubted that NPs may be replaced by other forms of nature protection. The differences between municipalities in general approval for NPs may be due to the different land use structure, however social and political factors might also play a role, such influence of the local forest inspectorates (Niedziałkowski et al. 2012). Ownership of a farm and professional relations with the logging industry were associated with a more negative approach towards NPs and professional relations with tourism were associated with a positive opinion, which reflects finding from other studies (Grodzińska-Jurczak and Cent 2011, Niedziałkowski et al. 2014, Kamal et al. 2015).

Our results support conclusion from other studies, that promoting PAs requires consideration of diversities within local communities and design of tailor-made communication and information activities that consider regional socioeconomic and political contexts (Karanth and Nepal 2012, Oldekop et al. 2015, MacKenzie et al. 2017). In case of the NPs, it may suggest site-specific governance, better integration of local communities into decision-making by using the knowledge of local stakeholders, accounting for people’s perceptions and integrating the demand for ES (Schirpke et al. 2021). Interestingly, the TuNP project has long included only state-owned land which theoretically should eliminate conflicts with most local private owners and entrepreneurs mitigating the negative local opinion towards the planned park. However, it did not prevent conflict and adverse opinions of local people. The conflict can be explained by the current governance model, as most of this area (both forest and non-forest locations) is managed by The State Forests, which derives significant profits from the forest management (RDLP Krosno 2017) and are the main economic beneficiary of local forest resources. Local forestry provides employment, actively participate in the life of local communities, cooperate with local entrepreneurs, having a significant impact on local public opinion (Boćkowski et al. 2020). Furthermore, representatives of local administration throughout Poland generally supports the State Forests in terms of their right to define the principles of nature protection in forest areas (Referowska-Chodak 2020). Polish local administration expects also that they will bear the financial consequences and resulting responsibility if forest governance was led by conservation institutions, such as national parks.

We also found a strong, positive correlation between the opinion towards the creation of the TuNP and the positive opinion on the current role of NPs in general. This implies that the strengthening of general views on PAs can contribute to improving local perceptions of PAs nearby, since, as previously shown, the former may explain the latter (Arnberger and Schoissengeier 2012, Job et al. 2021). This is also in line with the observation that PPR worldwide seem to be defined by residents’ personal opinions towards PAs (Allendorf 2022). Furthermore, the opinions towards NPs can be derivatives of people’s perceptions of negative and positive attributes of NPs, which can be, for example, “direct benefits”, such as resource extraction or cultural benefits (ibid.). In every studied municipality, general opinion towards NPs was significantly more positive than perception of the planned TuNP displaying a gap between these two. This might be partly a result of the locally manifested “not-in-my-backyard” (NIMBY syndrome), (Wexler 1996), which explains “locally unwanted land uses” (LULUs, Freudenburg and Pastor 1992). To better understand these mechanisms, the application of a multi-level PA conflict framework might be necessary (Rechciński et al. 2019). It is worth noting that a distrust as a source of opposition to NP may obscure economic assessments of benefits and disadvantages being commonly assumed as primary factors (Stern 2008). Hence, we would like to note that the discrepancy revealed may have a distant origin, first in forced resettlement after the World War II and second in the period of long-term isolation of the studied area for the state use by the then communist regime in Poland (Affek 2015). Such extreme examples of a top-down approach to landscape management could have resulted in a resistance to the authorities and centralized decisions that continue to this day; as in other post-socialist European countries, nature conservation is still often associated with state domination and command-and-control management (Kluvánková-Oravská et al. 2009, Yakusheva 2019). Therefore, it has been argued that the development of local community acceptance for a given PA is strongly dependent on the participation of residents in the decision-making process (Andrade and Rhodes 2012).

## Conclusions

This study has shown that using an ES lens can help to explore the factors that are important in the establishment and management of PAs, and it also suggests relevant areas to focus on when designing approaches to improve people’s opinions on PAs.

There are several implications for the results of the study for both the specific case of TuNP in the current legal and socioeconomic environment. First, the creation of an NP can have both negative and positive impacts on local communities, which are unequally distributed (Ward et al. 2018). The potential implications of conservation may affect various actors within, adjacent to and beyond park areas, so there is a need for predictive social impact assessment when planning the PA and further monitoring after its establishment (Kaplan-Hallam and Bennett 2017).

Second, the creation of a NP may also be considered in terms of reallocation of resource rights and benefits that create positive or negative changes in economy, health, education, and culture as well as other secondary social impacts. Therefore, such establishment should be preceded by several steps, beginning with identifying affected groups and ending with assessing the impact of possible rights reallocation (Kaplan-Hallam and Bennett 2017). For example, our findings suggest that gender as a control variable may play a role in shaping perceptions and approval towards the park, which could have implications for how outreach and engagement activities are designed and implemented. These insights could be instrumental in fostering a more harmonious relationship between the local inhabitants and the proposed TuNP.

Third, a comprehensive framework taking a NP as a socio-ecological system and using an ES approach can be applied to link the biophysical world to socioeconomic contexts. Such an approach should facilitate stakeholders’ involvement and reduce trade-offs between ES important for local livelihood and those of wider public importance (He et al. 2018b). While we agree NPs should remain a permanent element of the nature protection system, we argue that the role of NPs is not defined once and for all because they must adapt to the dynamically evolving challenges of the modern world. Establishing new NPs would require long-term political and financial commitment along with collaborative management (Andrade and Rhodes 2012) and deliverable approaches to address complex conservation problems (Raymond et al. 2013, Oliver et al. 2021).

Finally, ongoing in-depth studies of the stakeholder perceptions are then critical, as ultimately it is positive perceptions, not scientific evidence, that ensure local municipality support and long-term positive outcome of conservation efforts (Bennett 2016). More inter-, and transdisciplinary approaches are needed to better understand the complex relationships between stakeholders (Moon et al. 2019; Grodzińska-Jurczak et al. 2022), including system thinking, identifying key feedback loops driving system dynamics, and targeting leverage points transforming a system towards a desirable state (Schirpke et al. 2020).

The concept of ES can be used here as a ‘boundary object’ that uses interpretative flexibility to bring opposing stakeholders to the table (Steger et al. 2018), especially in countries that are reforming and consolidating their PA system (Mączka et al. 2019). We are aware of the limitations of our study in this respect, as we only focused on selected possible predictors of opinions towards NPs (see Job et al. 2021). Limitations of the ES approach include possible methodological inconsistencies between studies, problems with the precise wording of ES, the question of including intrinsic values as ES or the non-comparability character of results (Tusznio et al. 2020). When operationalizing of ES in the practice of establishing NPs, methodological, conceptual and policy facilitation challenges should be carefully considered (Carmen et al. 2018; Jax et al. 2018).

### Supplementary information

Annex No. 1

Annex No. 2

Annex No. 3

Annex No. 4

Annex No. 5

Annex No. 6

Annex No. 7

Annex No. 8

Annex No. 9
