# Supplementary material for: Ecosystem Services Approach in Turnicki National Park Planning: Factors Influencing the Inhabitants’ Perspectives on Local Natural Resources and Protected Areas
Source: Environ Manage. 2024 Jul 18;74(3):547–63. doi: 10.1007/s00267-024-02016-x (PMC11306527; doi:10.1007/s00267-024-02016-x)
Supplement: Supplementary file 1 — Annex No. 1 [file 267_2024_2016_MOESM1_ESM.docx]

Annex No. 1 Case study area description

| **DATA (as of 2019)** | **BIRCZA**  **MUNICIPALITY** | **FREDROPOL**  **MUNICIPALITY** | **USTRZYKI DOLNE**  **MUNICIPALITY** | **POLAND** |
| --- | --- | --- | --- | --- |
| Area (km^2^) | 254 | 160 | 479 | 312 705 |
| Population | 6 539 | 5 497 | 17 215 | 38 382 576 |
| Population density per km^2^ | 26 | 34 | 36 | 123 |
| Forest cover (%) | 61,2 | 54,4 | 61,4 | 29,6 |
| Public forests (%) | 92,7 | 93,3 | 93,7 | 76,7 |
|  |  |  |  |  |
| Unemployment (%) | 12,5*/8,9** | 12,5* / 7,9** | 13,5*/8,2** | 5,2*/3,4** |
| Gross salary in US $ | 996^***^ | 996^***^ | 1 124^***^ | 1 365^***^ |
| Migration | -25 | -43 | -81 | +6 183 |
|  |  |  |  |  |
| Users of network water supply system (% of population) | 14,4 | 8,7 | 74,1 | 92,2 |
| Users of sewage installation (% of population) | 35,9 | 41,2 | 49,7 | 71,2 |
| Users of network gas installation (% of population) | 4,3 | 5,3 | 0,5 | 52,9 |

Notes: * The registered unemployment rate is calculated based on share of registered unemployed persons to the economically active civilian population, i.e. excluding employees of budgetary entities conducting activity within the scope of national defence and public safety. Data is aggregated on the level of the ‘*powiat*’ (district), which is the primary administrative region referred to in Article 2(2)(p) of Directive 64/432/EEC. **Unemployment rate as a share of total working-age population.
***Based on middle exchange rates archive of the central bank of the Republic of Poland (Table No. 251/A/NBP/2019 of 2019-12-31). Data is aggregated on the level of the ‘*powiat*’ (district). Source: LDB 2019 (<https://bdl.stat.gov.pl/BDL/start>), SO Rzeszów 2020 (https://rzeszow.stat.gov.pl/en/statistical-vademecum-of-regional-civil-servant/).
