# Supplementary material for: Ecosystem Services Approach in Turnicki National Park Planning: Factors Influencing the Inhabitants’ Perspectives on Local Natural Resources and Protected Areas
Source: Environ Manage. 2024 Jul 18;74(3):547–63. doi: 10.1007/s00267-024-02016-x (PMC11306527; doi:10.1007/s00267-024-02016-x)
Supplement: Supplementary file 2 — Annex No. 2 [file 267_2024_2016_MOESM2_ESM.docx]

**Annex No. 2 fsQCA procedure description**

fsQCA is a methodological approach that allows for the examination of complex causal relationships and is particularly useful for identifying combinations of conditions that lead to a particular outcome (Ragin, 2008). Unlike traditional statistical methods that assume linear, additive relationships between variables, fsQCA allows for the exploration of non-linear, interactive relationships. It is based on set theory and Boolean algebra, and it allows for the identification of necessary and sufficient conditions for an outcome to occur (Ragin, 2000). One of the key advantages of fsQCA is its ability to handle asymmetric relationships. Traditional statistical methods assume that the relationship between a cause and an effect is symmetric, meaning that the presence of a cause leads to the presence of an effect, and the absence of a cause leads to the absence of an effect. However, in many real-world situations, relationships are asymmetric. For example, the presence of a cause may lead to the presence of an effect, but the absence of the cause does not necessarily lead to the absence of the effect. fsQCA allows for the modeling of such asymmetric relationships (Pappas & Woodside, 2021). In fsQCA, the key measures of a solution's quality are consistency and coverage. Consistency measures the degree to which a combination of conditions leads to an outcome. It is calculated as the proportion of cases with a given combination of conditions that have the outcome.

A consistency score close to 1 indicates that a combination of conditions almost always leads to the outcome, while a score close to 0 indicates that the combination rarely leads to the outcome. Coverage, on the other hand, measures the empirical importance of a solution. It is calculated as the proportion of all cases with the outcome that are covered by a given combination of conditions. A high coverage score indicates that a combination of conditions accounts for a large proportion of the cases with the outcome, while a low score indicates that the combination accounts for only a small proportion of these cases (Woodside 2013).

The formulas for consistency and coverage are as follows:

$$Consistency= \sum\frac{\min(x_{i}, y_{i})}{\sum(x_{i})}$$

$$Coverage= \sum\frac{\min(x_{i}, y_{i})}{\sum(y_{i})}$$

where x_i_ is the membership score of a case in the condition set, and y_i_ is the membership score of a case in the outcome set.

Literature:

Pappas IO, Woodside AG (2021) Fuzzy-set Qualitative Comparative Analysis (fsQCA): Guidelines for research practice in Information Systems and marketing. International Journal of Information Management, 58: 102310

Ragin CC (2008) Measurement versus calibration: A set‐theoretic approach

Ragin CC (2000) Fuzzy-set social science. University of Chicago Press

Woodside AG (2013) Moving beyond multiple regression analysis to algorithms: Calling for adoption of a paradigm shift from symmetric to asymmetric thinking in data analysis and crafting theory. Journal of Business Research 66(4): 463-472.
