# Supplementary material for: Ecosystem Services Approach in Turnicki National Park Planning: Factors Influencing the Inhabitants’ Perspectives on Local Natural Resources and Protected Areas
Source: Environ Manage. 2024 Jul 18;74(3):547–63. doi: 10.1007/s00267-024-02016-x (PMC11306527; doi:10.1007/s00267-024-02016-x)
Supplement: Supplementary file 3 — Annex No. 3 [file 267_2024_2016_MOESM3_ESM.docx]

Annex No. 3 Choice of major types of services groups with different attitudes towards TuNP

| ATTITUDE OF RESPONDENTS |  | PROVISIONAL | CULTURAL | | REGULATORY | |
| --- | --- | --- | --- | --- | --- | --- |
| negative/rather negative | Count | 505 | | 214 | | 183 |
|  | % | 56% | | 24% | | 20% |
| neutral | Count | 228 | | 162 | | 107 |
|  | % | 46% | | 33% | | 22% |
| positive/rather positive | Count | 217 | | 132 | | 124 |
|  | % | 46% | | 28% | | 26% |
