# Supplementary material for: Ecosystem Services Approach in Turnicki National Park Planning: Factors Influencing the Inhabitants’ Perspectives on Local Natural Resources and Protected Areas
Source: Environ Manage. 2024 Jul 18;74(3):547–63. doi: 10.1007/s00267-024-02016-x (PMC11306527; doi:10.1007/s00267-024-02016-x)
Supplement: Supplementary file 4 — Annex No. 4 [file 267_2024_2016_MOESM4_ESM.docx]

Annex No. 4 Detailed choice of benefits in respondents who chose “negative” and “rather negative” in terms of support for TuNP

Notes: shades of red / orange indicate benefits classified as provisional ecosystem services, shades of blue - cultural services, and shades of green - regulatory services; data label percentages have been rounded to the nearest whole number; number of respondents: 1st choice N = 184, 2nd choice N = 185, 3rd choice N = 184, 4th choice N =177, 5th choice N = 167.
