# Supplementary material for: Ecosystem Services Approach in Turnicki National Park Planning: Factors Influencing the Inhabitants’ Perspectives on Local Natural Resources and Protected Areas
Source: Environ Manage. 2024 Jul 18;74(3):547–63. doi: 10.1007/s00267-024-02016-x (PMC11306527; doi:10.1007/s00267-024-02016-x)
Supplement: Supplementary file 6 — Annex No. 6 [file 267_2024_2016_MOESM6_ESM.docx]

Annex No. 6 Detailed choice of benefits in respondents who chose “positive” and “rather positive” in terms of support for TuNP

Notes: shades of red / orange indicate benefits classified as provisional ecosystem services, shades of blue - cultural services, and shades of green - regulatory services; data label percentages have been rounded to the nearest whole number; number of respondents: 1st choice N = 77, 2nd choice N = 77, 3rd choice N = 77, 4th choice N = 75, 5th choice N = 73. Source: own results.
