# Supplementary material for: Ecosystem Services Approach in Turnicki National Park Planning: Factors Influencing the Inhabitants’ Perspectives on Local Natural Resources and Protected Areas
Source: Environ Manage. 2024 Jul 18;74(3):547–63. doi: 10.1007/s00267-024-02016-x (PMC11306527; doi:10.1007/s00267-024-02016-x)
Supplement: Supplementary file 7 — Annex No. 7 [file 267_2024_2016_MOESM7_ESM.docx]

Annex No. 7 Influence of sociodemographic and economic factors on support for national parks and the planned Turnicki National Park

| **VARIABLE** | **NATIONAL PARKS** | **PLANNED TURNICKI NP** |
| --- | --- | --- |
| Age of the respondents | 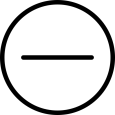 | 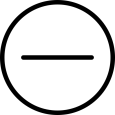 |
| Number of years of living in the municipality | 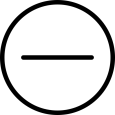 | 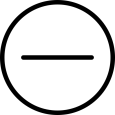 |
| Level of education | 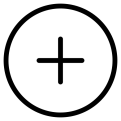 | 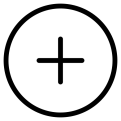 |
| Average monthly net income | 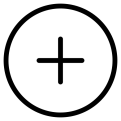 | 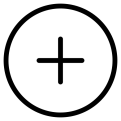 |
| Opinion on national parks | N/A | 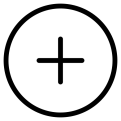 |
| Opinion toward planned Turnicki NP | 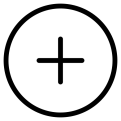 | N/A |
|  |  |  |
| Number of people in a household | **🗴** | **🗴** |
| Respondents’ gender | **🗴** | **🗴** |
| Occupational situation | **?** | **🗴** |
| Having a farm | 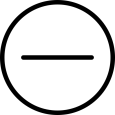 | **🗴** |
| Incomes related to tourism | 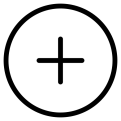 | 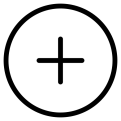 |
| Incomes related to logging industry | 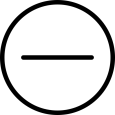 | **🗴** |

Note: Icons mark negative (red) and positive (green) correlations (upper part) or relationships (lower part) between the variables. Darker color marks a strong correlation. A cross means there was no demonstrated statistically significant relationship between the variables. A question mark means an ambiguous result.
