# Supplementary material for: Ecosystem Services Approach in Turnicki National Park Planning: Factors Influencing the Inhabitants’ Perspectives on Local Natural Resources and Protected Areas
Source: Environ Manage. 2024 Jul 18;74(3):547–63. doi: 10.1007/s00267-024-02016-x (PMC11306527; doi:10.1007/s00267-024-02016-x)
Supplement: Supplementary file 8 — Annex No. 8 [file 267_2024_2016_MOESM8_ESM.docx]

Annex No. 8 Effect of type of economic activity on opinions on national parks

|  | **Do you have farm?** | | **N** | **Mean** | | **SD** | | **Std. Error** | **t** | **df** | **Sig. (two-tailed)** |
| --- | --- | --- | --- | --- | --- | --- | --- | --- | --- | --- | --- |
| **Approval for NPs** | No | | 246 | 3,29 | | .92211 | | .05879 | 3.911 | 522.981 | .000* |
|  | Yes | | 279 | 2,95 | | 1.05240 | | .06301 |  |  |  |
| **Approval for Turnicki NP** | No | | 158 | 2,61 | | 1.382 | | .110 | 1.958 | 364 | .051 |
|  | Yes | | 208 | 2,32 | | 1.423 | | .099 |  |  |  |
|  | **Are your incomes related to tourism?** | | **N** | **Mean** | | **SD** | | **Std. Error** | **t** | **df** | **Sig. (two-tailed)** |
| **Approval for NPs** | No | | 482 | 3,08 | | .99126 | | .04515 | -2.059 | 523 | .040* |
|  | Yes | | 43 | 3,41 | | 1.13571 | | .17319 |  |  |  |
| **Approval for Turnicki NP** | No | | 332 | 2,39 | | 1.390 | | .076 | -2.175 | 364 | .030* |
|  | Yes | | 34 | 2,94 | | 1.536 | | .263 |  |  |  |
|  | **Are your incomes related to logging industry?** | | **N** | **Mean** | | **SD** | | **Std. Error** | **t** | **df** | **Sig. (two-tailed)** |
| **Approval for NPs** | No | | 414 | 3,19 | | .99996 | | .04915 | 3.370 | 523 | .001* |
|  | Yes | | 111 | 2,83 | | .98541 | | .09353 |  |  |  |
| **Approval for Turnicki NP** | No | | 277 | 2,52 | | 1.418 | | .085 | 1.767 | 364 | .078 |
|  | | Yes | | 89 | 2,21 | | 1.369 | .145 |  |  |  |
