# Supplementary material for: Ecosystem Services Approach in Turnicki National Park Planning: Factors Influencing the Inhabitants’ Perspectives on Local Natural Resources and Protected Areas
Source: Environ Manage. 2024 Jul 18;74(3):547–63. doi: 10.1007/s00267-024-02016-x (PMC11306527; doi:10.1007/s00267-024-02016-x)
Supplement: Supplementary file 9 — Annex No. 9 [file 267_2024_2016_MOESM9_ESM.docx]

Annex No. 9 Results of two-way Anova test for limitations and threats to benefits versus approval of national parks and opinion towards Turnicki NP

|  | | | | | | |
| --- | --- | --- | --- | --- | --- | --- |
| Dependent variable: Approval of national parks  Independent variables: B. 2 Current limitations of benefits / B. 5 Threats for benefits from nature | | | | | | |
| Source | Type III sum of squares | df | Mean square | F | Sig. | partial Eta squared |
| Corrected model | 6.013^a^ | 3 | 2.004 | 1.982 | .116 | .011 |
| Intercept | 3504.150 | 1 | 3504.150 | 3465.099 | .000 | .869 |
| B.2 | .183 | 1 | .183 | .181 | .671 | .000 |
| B.5 | 4.373 | 1 | 4.373 | 4.325 | .038* | .008 |
| B.2 * B5 | .407 | 1 | .407 | .402 | .526 | .001 |
| Error | 527.883 | 522 | 1.011 |  |  |  |
| Total | 5639.078 | 526 |  |  |  |  |
| Corrected total | 533.896 | 525 |  |  |  |  |
| a. R squared = .011 (Adjusted R squared = .006) | | | | | | |

|  | | | | | | |
| --- | --- | --- | --- | --- | --- | --- |
| Dependent variable: Attitude towards Turnicki NP  Independent variables: B. 2 Current limitations benefits / B. 5 Threaths for benefits from nature | | | | | | |
| Source | Type III sum of squares | df | Mean square | F | Sig. | partial Eta squared |
| Corrected model | 12.091^a^ | 3 | 4.030 | 2.043 | .107 | .017 |
| Intercept | 1404.101 | 1 | 1404.101 | 711.793 | .000 | .663 |
| B.2 | 3.181 | 1 | 3.181 | 1.612 | .205 | .004 |
| B5 | 4.423 | 1 | 4.423 | 2.242 | .135 | .006 |
| B.2 * B5 | .186 | 1 | .186 | .094 | .759 | .000 |
| Error | 712.117 | 361 | 1.973 |  |  |  |
| Total | 2909.000 | 365 |  |  |  |  |
| Corrected total | 724.208 | 364 |  |  |  |  |
| a. R squared= .017 (Adjusted R squared = .009) | | | | | | |
